# Deuteron‐Decoupled Singlet NMR in Low Magnetic Fields: Application to the Hyperpolarization of Succinic Acid**

**DOI:** 10.1002/cphc.202200274

**Published:** 2022-08-04

**Authors:** Laurynas Dagys, Christian Bengs, Gamal A. I. Moustafa, Malcolm H. Levitt

**Affiliations:** ^1^ School of Chemistry Highfield Campus Southampton SO17 1BJ United Kingdom

**Keywords:** spin decoupling, low-field NMR, nuclear singlet states, para-hydrogen, singlet-triplet mixing

## Abstract

The reaction of unsaturated substrates with hydrogen gas enriched in the *para* spin isomer leads to products with a high degree of nuclear singlet spin order. This leads to greatly enhanced NMR signals, with important potential applications such as magnetic resonance imaging (MRI) of metabolic processes. Although parahydrogen‐induced polarization has the advantage of being cheap, compact, and mobile, especially when performed in ultralow magnetic fields, efficiency is lost when more than a few protons are involved. This strongly restricts the range of compatible substances. We show that these difficulties may be overcome by a combination of deuteration with the application of a sinusoidally modulated longitudinal field as a well as a transverse rotating magnetic field. We demonstrate a six‐fold enhancement in the ^13^C hyperpolarization of [1‐^13^C, 2,3‐d_2_]‐succinic acid, as compared with standard hyperpolarization methods, applied in the same ultralow field regime.

## Graphical abstract

**The combination of para‐hydrogen and low‐field techniques** allows efficient technical solutions using weak rotating and oscillating magnetic fields that provides a compact and rapid method for NMR hyperpolarization.

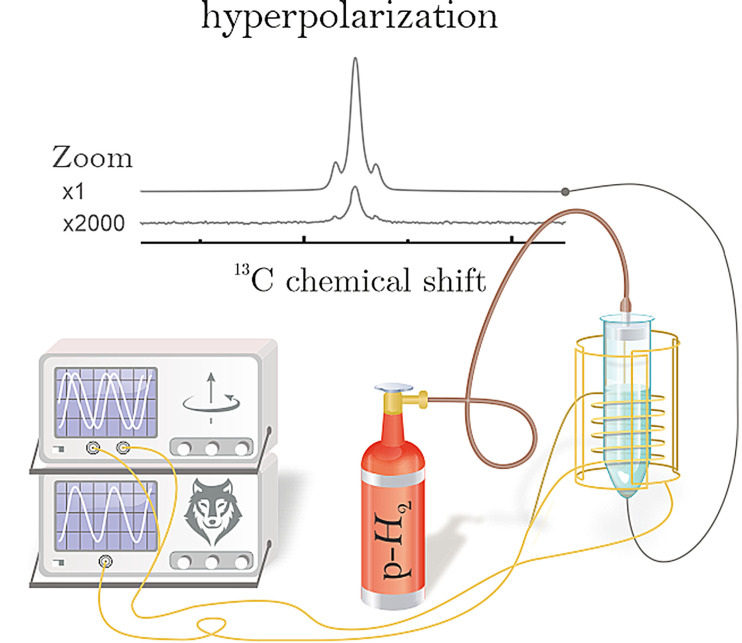

## Introduction

**Figure 1:**
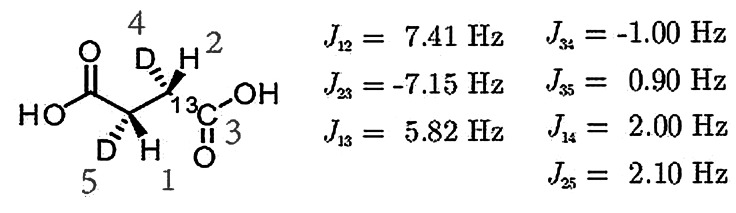
Chemical structure of [1‐^13^C, 2,3‐d_2_]‐succinic acid. The spin‐spin couplings have been reported previously,^[19]^ except for those involving deuterium, which are estimated. The labile protons are ignored.

Magnetic Resonance Imaging (MRI) represents a versatile, non‐invasive diagnostic tool, but suffers from low sensitivity, due in part to the very low levels of equilibrium nuclear spin polarization. Very large (∼10^5^) polarization enhancements are available by hyperpolarization techniques,[^1^, ^2^, ^3^, ^4^, ^5^, ^6^, ^7^, ^8^, ^9^, ^10^] leading to the possibility of imaging low‐concentration metabolites, with important clinical applications.[^1^, ^2^, ^3^, ^4^, ^5^, ^6^, ^7^, ^8^] Hyperpolarization is particularly advantageous for ^13^C‐labelled substances, since ^13^C nuclei often have relatively long relaxation times compared to the abundant protons, allowing the hyperpolarization effect to persist longer, as well as a large chemical shift range, facilitating compound‐specific imaging. For example, hyperpolarization of ^13^C‐labelled metabolites such as pyruvate, fumarate and succinate (1,4‐butanedioic acid, see Figure 1) allow monitoring of the tricarboxylic acid (TCA) cycle, exposing metabolic anomalies which are indicative of disorders such as cancer.[^2^, ^3^, ^4^, ^5^, ^6^, ^7^, ^8^, ^9^, ^10^]

Most applications of hyperpolarized compounds for *in vivo* metabolic imaging currently use the Dynamic Nuclear Polarization (DNP) technique, for which commercial apparatus exists.[^1^, ^2^, ^3^, ^4^, ^5^, ^6^, ^7^, ^10^] However, the DNP technique suffers from high capital and running costs, low throughput, large footprint and technical complexity. This has stimulated interest in compact, mobile and less expensive methods such as parahydrogen‐induced polarization (PHIP).[^11^, ^12^] Although less general than DNP, PHIP‐based techniques do not require high magnetic fields or cryogenic equipment, and also have high potential throughput.

Parahydrogen‐induced polarization of a ^13^C‐labelled target substance is achieved by catalytic hydrogenation of a suitable precursor with *para*‐enriched hydrogen gas (p*H*_2_). The proton pairs in the reaction product acquire a high degree of nuclear singlet order. The non‐magnetic nuclear singlet order is converted into observable ^13^C magnetization by the application of suitably modulated magnetic fields, leading to large enhancements of the ^13^C NMR signals.[^13^, ^14^, ^15^, ^16^, ^17^, ^18^]

The conversion of proton singlet order into ^13^C magnetization is a crucial step. Methods exist for implementing this transformation either in the high magnetic field of an NMR magnet, or in low magnetic field. Low‐field procedures are preferred for real‐world applications, since they allow the use of relatively inexpensive, compact, and mobile equipment.[^20^, ^21^, ^22^, ^23^, ^24^] Recently, purified solutions of highly polarized [1‐^13^C]‐fumarate have been produced using a low magnetic field sweep for singlet‐to‐magnetization conversion.^[24]^ Polarized [1‐^13^C]‐fumarate is a favourable case since it has a very simple spin system, containing only three coupled spins‐1/2 with long relaxation times.

The application of such procedures to more complex spin systems is problematic. Every additional spin doubles the spin dynamical complexity and decreases the transformation efficiency.[^19^, ^25^, ^26^] For example, [1‐^13^C]‐succinate contains 5 coupled spins‐1/2, and has 4 times as many quantum states as [1‐^13^C]‐fumarate. In general field‐sweep methods perform poorly for multiple‐spin systems, since it is hard to maintain control of the highly complex nuclear spin dynamics, especially through the multiple level crossings associated with field sweeps.^[22]^

The number of coupled protons may be reduced by selective chemical replacement of protons by deuterons. An example is shown for [1‐^13^C, 2,3‐d_2_]‐succinic acid in Figure 1. Although this leads to a beneficial simplification of the proton spin system, deuteration introduces problems in the context of ultralow‐field NMR, where the spin‐spin couplings are of the same order of magnitude as nuclear Larmor frequencies. In this regime, the rapid quadrupolar relaxation of the deuterium nuclei induces rapid decoherence of the entire spin system, resulting in substantial polarisation losses.[^27^, ^28^, ^29^, ^30^] Furthermore, singlet‐triplet mixing effects induced by heteronuclear couplings lead to further losses.[^14^, ^31^, ^32^, ^33^, ^34^] Conventional heteronuclear decoupling is inapplicable in the ultralow‐field regime, since the nuclear Larmor frequencies are not sufficiently distinct. In order to fully leverage parahydrogen‐induced polarization in the microtesla regime, polarization transfer strategies are required that not only decouple disruptive deuterium nuclei, but also suppress singlet‐triplet mixing effects, in the regime of ultralow magnetic fields.

An alternative approach is to perform experiments in milliTesla fields, where the Larmor frequencies of different isotopes are sufficiently distinct to allow selective heteronuclear decoupling by conventional resonant irradiation. However, although the use of milliTesla fields is certainly feasible,^[8]^ it is associated with technical challenges such as the stabilization of the field, and the use of relatively bulky equipment such as permanent magnets.

In this article we demonstrate efficient singlet‐to‐mag‐netization conversion in the microTesla regime, while simultaneously achieving deuterium decoupling and suppression of singlet‐triplet mixing. This is done by applying two periodically modulated fields at the same time. The first field is a sinusoidally modulated longitudinal field, as in the recently described Weak Oscillating Low Field (WOLF) technique.^[35]^ The second field rotates in a plane perpendicular to the first, as in the recently described Singlet‐Triplet Oscillations through Rotating Magnetic Fields (STORM) method.^[36]^ We show that the combination of these two fields achieves selective polarization transfer with suppression of deuterium‐induced relaxation. We achieve a ^13^C polarization of ≃6.1 % for a 50 mM solution of [1‐^13^C, 2,3‐d_2_]‐succinic acid, in the case of a modest parahydrogen enrichment level of 50 %. This result is encouraging for the application of low‐field parahydrogen‐induced polarization to a wide range of deuterated molecular systems.

## Experimental Section

### Materials and Equipment

To demonstrate the technique, 30 mg (0.25 mmol) of [1‐^13^C, 2,3‐d_2_]‐fumaric acid (Sigma–Aldrich) and 18.1 mg (0.025 mmol) of the [Rh(dppb)(COD)]BF_4_ catalyst were dissolved in methanol‐d_4_ (5 mL). 50 %‐*para*‐enriched H_2_ was prepared by passing hydrogen gas at a pressure of 10 bar over iron oxide at 77 K. Each experimental run started by transferring 250 *μ*L of the stock solution to a high‐pressure NMR tube and heating the sample to 75 °C in the ambient magnetic field produced by the magnet (110 *μ*T). The heated sample was transferred to a magnetically shielded chamber (three concentric walls of mu‐metal, from Twinleaf LLC, USA) equipped with magnetic field coils. *Para*‐enriched hydrogen gas was dissolved in the solution by bubbling. This initiated the catalytic hydrogenation of the [1‐^13^C, 2,3‐d_2_]‐fumaric acid precursor, generating the singlet‐polarized target substance [1‐^13^C, 2,3‐d_2_]‐succinic acid. As discussed below, several methods were evaluated for the conversion of the proton singlet order into hyperpolarized ^13^C magnetization. Immediately after singlet‐to‐magnetization conversion, the level of ^13^C polarization was assessed by rapidly inserting the sample into a conventional 9.41 T NMR magnet, followed by generation of the ^13^C NMR spectrum by applying a single *π*/2 pulse and Fourier transformation of the free‐induction decay. Further details of the experimental implementation are given in the supplementary information.

### Experimental Protocols

**Figure 2:**
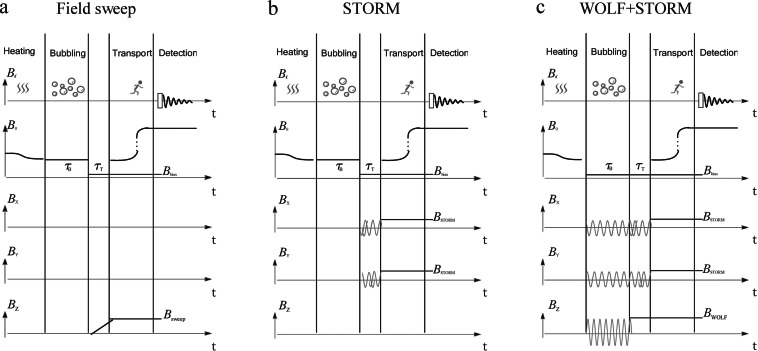
Timing diagrams for (a) hydrogenation followed by a conventional field sweep for polarization transfer; (b) hydrogenation followed by polarization transfer using a STORM pulse; (c) hydrogenation under simultaneous WOLF+STORM fields, followed by polarization transfer using a STORM pulse. In (a) *para*‐enriched hydrogen is bubbled through the sample for a duration *τ*_B_ in the presence of a static bias field (*B*_0_) along the laboratory frame z‐axis. During the period *τ*_T_ the field is linearly increased from 0 *μ*T to a target value *B*_sweep_. In (b) the magnetic field cycling is replaced by the application of a rotating magnetic field generated by two orthogonal transverse coils. The amplitude of the transverse rotating field is given by *B*_STORM_. In (c) the bubbling period is conducted under the simultaneous application of a small bias field *B*_bias_, a transverse rotating field of amplitude *B*_STORM_ and an oscillating longitudinal field of amplitude *B*_WOLF_. The singlet‐to‐magnetization polarization transfer is initiated by turning off the longitudinal WOLF field, while retaining the rotating transverse STORM field. In all three cases, the sample is transferred to a high‐field NMR apparatus immediately after the polarization transfer, followed by a *π*/2 pulse and signal acquisition on the ^13^C channel.

Figure 2 shows the three alternative protocols that were evaluated: (a) a conventional magnetic field cycling procedure for magnetization‐to‐singlet transformation;[^13^, ^22^] (b) a STORM procedure for the magnetization‐to‐singlet transformation, suppressing the effects of deuteron relaxation; (c) magnetization‐to‐singlet transformation by STORM, combined with suppression of singlet‐triplet mixing during hydrogenation by simultaneous application of STORM and WOLF.

Protocol (a) in Figure 2 uses a conventional magnetic field cycling procedure for singlet‐to‐magnetization transfer.[^13^, ^22^] This was performed as follows: *Para*‐enriched H_2_ was bubbled through the solution for an interval of duration *τ*_B_, in the presence of a static bias field of 50 *μ*T, in order to separate the Larmor frequencies of the different isotopes, helping preserve the proton singlet order. The magnetic field was quickly reduced to 0 T and then swept linearly from ≃0 *μ*T to ≃1 *μ*T over a duration *τ*_T_, to induce the singlet‐to‐magnetization transfer. As shown in Table 1, experimental optimization of the bubbling time (*τ*_B_=7 s) and the transfer time (*τ*_T_=90 ms) resulted in a ^13^C polarization of *p*=0.98 %±0.04 % for the [1‐^13^C, 2,3‐d_2_]‐succinic acid reaction product. A representative spectrum for protocol (a) is given in Figure 2a. The moderate level of ^13^C polarization level is not surprising, since the conventional magnetic field cycling protocol is subject to interference from both deuterium quadrupolar relaxation and singlet‐triplet mixing.

**Table 1:** Experimentally determined ^13^C polarization levels of [1‐^13^C, 2,3‐d_2_]‐succinic acid for protocols (a), (b) and (c) in Figure 2, using the indicated bubbling time *τ*_B_ and transfer time *τ*_T_, which were optimized for each protocol individually.

Method	*τ*_B_	*τ*_T_	Polarization level
Field sweep	7 s	90 ms	0.98 %±0.04 %
STORM	7 s	100 ms	1.70 %±0.22 %
WOLF+STORM	15 s	100 ms	6.13 %±0.07 %

**Figure 3:**
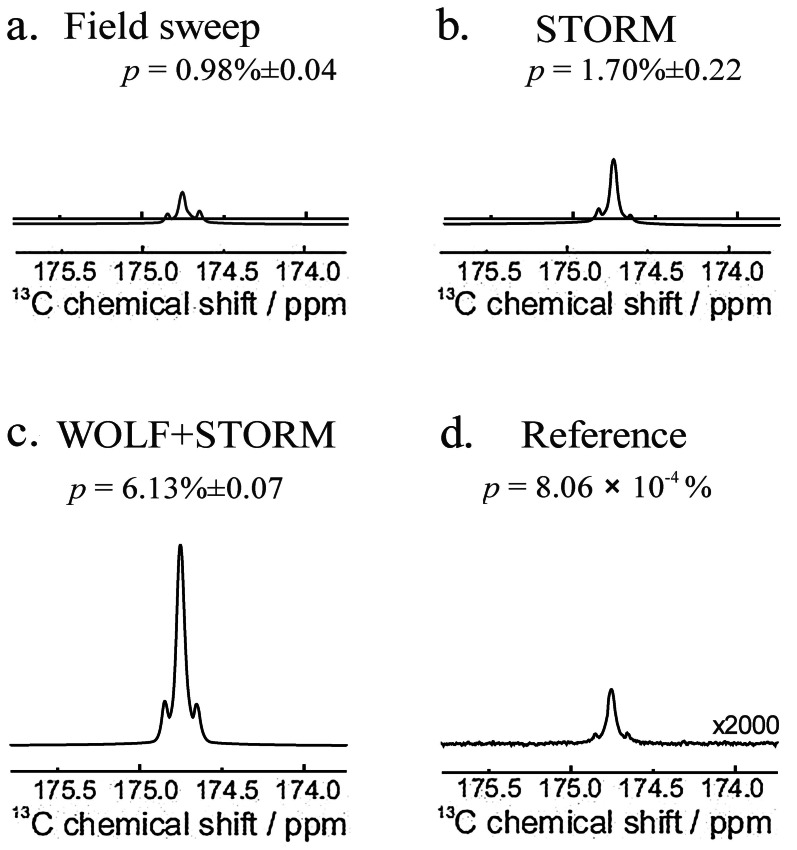
Hyperpolarized ^13^C spectra of [1‐^13^C, 2,3‐d_2_]‐succinic acid acquired at 9.41 T, obtained by using the protocols in Figure 2. (a) Polarization transfer induced by a 90 ms duration magnetic field sweep from 0 to 1 *μ*T. (b) STORM polarization transfer at bias field *B*_bias_≃6 *μ*T with rotation frequency *ω*STORM=227 Hz and amplitude *B*_STORM_=3.9 *μ*T. (c) Application of WOLF+STORM fields during bubbling with a WOLF amplitude *B*_WOLF_=20 *μ*T and frequency *ω*_WOLF_=800 Hz, simultaneous with a STORM field with the same parameters as in (b), and followed by polarization transfer using STORM. (d) Conventional ^13^C NMR spectrum obtained on a thermal equilibrium sample (averaged over 32 transients). Estimated ^13^C polarization levels are indicated.

Protocol (b) in Figure 2 employs the rotating‐field STORM technique^[36]^ for the polarization transfer. After bubbling with *para*‐enriched H_2_, the transverse rotating STORM field was applied for a duration *τ*_T_ in the presence of a longitudinal 6 *μ*T bias field. The amplitude of the rotating STORM field was *B*_STORM_=3.9 *μ*T. The rotation frequency of the STORM field was *ω*_STORM_/2*π*=227 Hz. As discussed in the supplementary material, this choice of field parameters decouples the proton spins from the deuterium spins during the transfer step. Figure 3b shows the resulting ^13^C spectrum for an optimal STORM duration of *τ*_T_=100 ms. The final ^13^C polarization was estimated to be *p*=1.70 %±0.22 %, which is significantly higher than that for protocol (a). Since the bubbling protocols are identical in procedures (a) and (b), we attribute the increase in the ^13^C polarization level to the robustness of the STORM pulse with respect to interference from fast deuterium relaxation.

Although the use of a rotating‐field STORM suppresses the deleterious effects of deuteron relaxation during the singlet‐to‐magnetization transfer, the problem of singlet‐to‐triplet mixing remains. In the current context of very low magnetic fields, this mixing is caused by differences in the J‐couplings of the two protons to heteronuclei such as ^13^C and ^2^H (see the coupling network in Figure 1). As a result of these couplings, proton singlet state is not a Hamiltonian eigenstate for the succinic acid product. Reaction of the fumaric acid precursor with *para*‐hydrogen therefore initiates coherent oscillations of the spin density operator. Since the hydrogenation reaction is relatively slow, these coherent oscillations destructively interfere over the hydrogenation interval, leading to significant polarization loss.

Protocol (c) in Figure 2 tests this hypothesis by applying simultaneous WOLF and STORM pulses during the hydrogenation interval, in order to suppress singlet‐triplet mixing in the product molecules. At first this seems counter‐intuitive, since a lone STORM pulse induces singlet‐to‐magnetization polarization transfer from the proton pair to the ^13^C nucleus, as demonstrated in protocol (b). Such a process would be undesirable during the slow hydrogenation reaction, since the premature transfer of singlet order to ^13^C magnetization would lead to a loss of spin order, when summed over all reacting molecules. However, as discussed in the supplementary material, the superposition of a resonant STORM pulse with a suitably chosen WOLF pulse suspends the polarization transfer until the hydrogenation is complete, while preserving the deuterium‐decoupling properties of the STORM pulse, and also suppressing singlet‐triplet mixing effects during hydrogenation.

For the experimental implementation of protocol (c) we kept the STORM pulse parameters identical to protocol (b), but added a WOLF pulse with amplitude *B*_WOLF_=20 *μ*T and frequency *ω*_WOLF_/2*π*=800 Hz during the hydrogenation interval. The WOLF pulse parameters were based on the analysis given in the supplementary information. The p*H*_2_ bubbling period was optimized to *τ*_B_=15 s for protocol (c). The resulting ^13^C spectrum is shown in Figure 3c, which corresponds to a ^13^C polarization of *p*=6.13 %±0.07 %. As highlighted in Table 1, this represents a factor of 6 improvement over a conventional field sweep procedure.

## Results and Discussion

The *molar polarization* (product of polarization level and concentration) has been proposed as a more useful hyperpolarization metric than the polarization level alone.^[24]^ The molar polarization achieved in the current experiment is estimated to be 6.12 %×(49±1) mM=3.00±0.06 mM (see Supporting Information).

**Figure 4:**
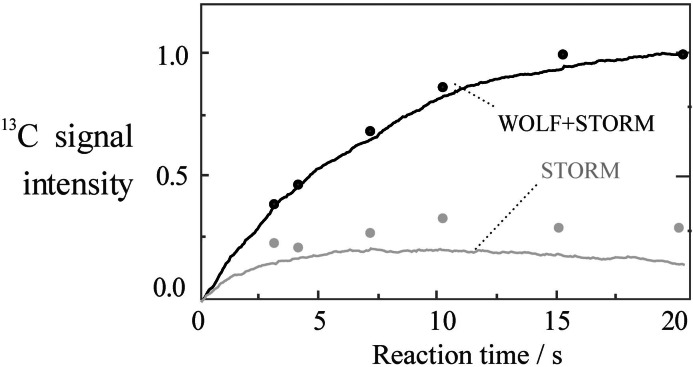
^13^C signal intensity of [1‐^13^C, 2,3‐d_2_]‐succinic acid as function of the reaction time *τ*_B_ for protocol (b) (grey lines) and protocol (c) (black lines). Solid lines show numerically simulated trajectories, whereas dotted points represent experimentally determined values. The vertical axis has been normalized to the maximum signal of the time series. Details of the simulation are given in the supplement.

Figure 4 compares the observed ^13^C‐polarization as a function of bubbling time *τ*_B_, for protocols (b) and (c). Since these protocols use the same singlet‐to‐magnetization conversion procedures, differences in performance may be attributed to the chemical and spin dynamics during the hydrogenation. The markers represent experimental data, whereas the solid curves represent numerical simulations. For protocol (b), which has no special intervention during the hydrogenation, the ^13^C‐polarization quickly reaches a plateau at a relatively low level.

For the WOLF+STORM approach (protocol (c)) the build‐up of ^13^C‐polarization continues to longer times with a plateau reached at *τ*_B_=15 s. No significant reduction in ^13^C‐polarization was observed even beyond 20 seconds of bubbling. These data support the hypothesis that the simultaneous WOLF+STORM fields suppress singlet‐triplet mixing during hydrogenation.

The solid lines in Figure 4 show simulations derived by a Monte‐Carlo procedure which combined many spin dynamical trajectories initiated at random times, according to a statistical model of the chemical kinetics. Details are given in the Supporting Information. An acceptable match between experiment and simulation is attained for both protocols (b) and (c), assuming an effective hydrogenation rate constant given by *k_eff_* =0.05±0.01 s^−1^ and a singlet order decay time constant *T_S_* of at least 60 seconds. This indicates that singlet spin order accumulates on the proton pair during the hydrogenation under WOLF+STORM irradiation.

## Conclusion

To summarize, we have demonstrated that it is possible to hyperpolarize the ^13^C nuclei of isotopically labelled succinate using a compact, low‐cost apparatus which exploits *para*‐enriched hydrogen gas, a cheap and readily available substance. The achieved level of ∼6 % ^13^C‐polarization could readily be increased to ∼18 % by using pure *para*‐H_2_ gas. This polarization level is competitive with dissolution‐DNP procedures but is achieved far more rapidly and with much less cost. This suggests the feasibility of this method for producing hyperpolarized ^13^C‐succinate for metabolic imaging applications. Further improvements in the yield and throughput are likely to be possible, for example by operation at higher pressure, temperature and optimised solvent and catalyst system.[^8^, ^24^]

The demonstration described here combines several chemical and spin dynamical methodologies: (1) the use of a deuterated and ^13^C‐labelled fumarate precursor, in order to generate a deuterated succinate with a simplified proton spin system; (2) novel magnetic field modulation methodologies applied under ultralow‐field conditions, which induce efficient singlet‐to‐magnetization transfer even in the presence of the rapidly relaxing deuterium nuclei, while also suppressing singlet‐triplet mixing effects during the hydrogenation.

We anticipate that this proof‐of‐concept experiment should open up the technique of ultralow‐field parahydrogen‐induced polarization to a much wider range of compounds than is currently accessible. Related hyperpolarization techniques such as side‐arm hydrogenation[^37^, ^38^] are also likely to benefit, allowing the use of deuteration to simplify the spin systems and improve spin‐dynamical efficiency, while avoiding the problems associated with rapid deuterium relaxation.

## Conflict of interest

There are no conflicts of interest to declare.

1

## Supporting information

As a service to our authors and readers, this journal provides supporting information supplied by the authors. Such materials are peer reviewed and may be re‐organized for online delivery, but are not copy‐edited or typeset. Technical support issues arising from supporting information (other than missing files) should be addressed to the authors.

Supporting Information

## Data Availability

The data that support the findings of this study are available from the corresponding author upon reasonable request.
