# Supplementary material for: Deuteron‐Decoupled Singlet NMR in Low Magnetic Fields: Application to the Hyperpolarization of Succinic Acid
Source: Chemphyschem. 2022 Aug 4;23(19):e202200274. doi: 10.1002/cphc.202200274 (PMC9804268; doi:10.1002/cphc.202200274)
Supplement: Supplementary file 1 — Supporting Information [file CPHC-23-0-s001.pdf]

ChemPhysChem

Supporting Information

Deuteron-Decoupled Singlet NMR in Low Magnetic Fields: Application to the Hyperpolarization of Succinic Acid**

Laurynas Dagys[+], Christian Bengs[+], Gamal A. I. Moustafa, and Malcolm H. Levitt*

Experimental setup

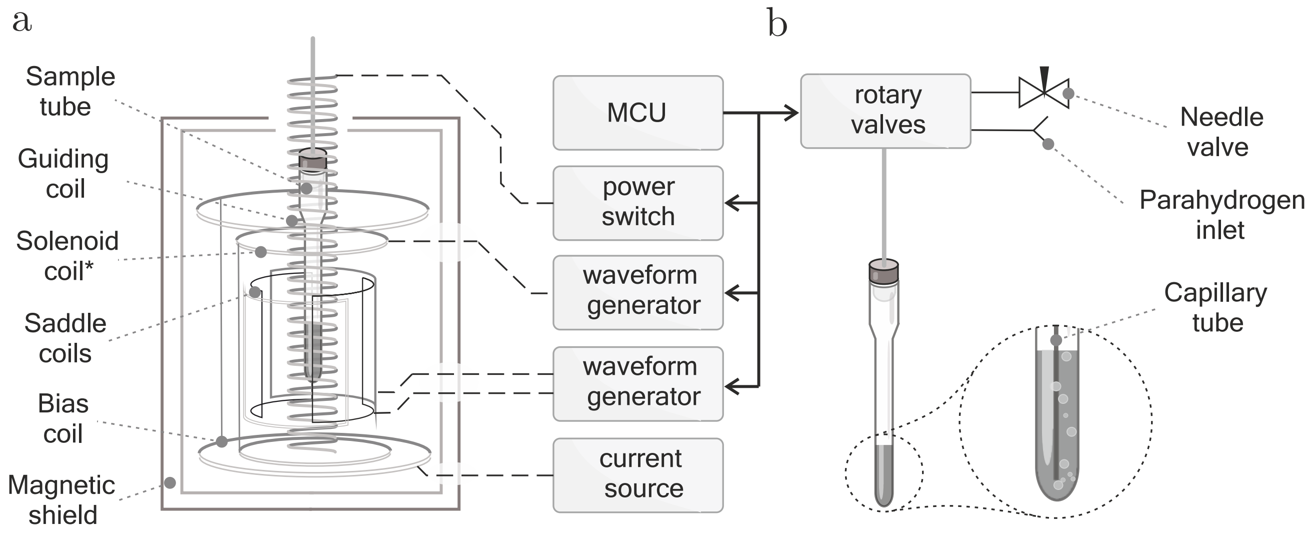

Figure S1 Schematic overview of the experimental setup. (a) Twinleaf Mu-metal shield with its additional components. The Twinleaf Helmholtz coil (bias coil) generates a bias field of strength B_{bias}, whereas two saddle coils generate a rotating magnetic field of strength B_{STORM}. The solenoid coil (not explicitly depicted) provides an oscillating magnetic field of strength B_{STORM}. The guiding coil generates a 50 μT field during removal of the sample. (b) Gas-flow control strategy. A high-pressure NMR tube is equipped with a capillary for bubbling of pressurised *para*-enriched H_2 gas. The rotary valves are actuated using a micro-controller unit (MCU). *Solenoid coil is illustrated by two rings for simplicity.

A schematic overview of the experimental equipment is shown in figure S1. The hydrogen gas is added to the solution using a 1/16" PEEK capillary tube inserted into a thin-walled Norell® high-pressure NMR tube. A 40 cm long and 15 mm wide coil was designed to provide a 50 μT guiding field piercing through the TwinLeaf MS-4 mu-metal shield providing a guiding field as the sample is removed. A 12 cm long and 5 cm wide coil was designed to generate an oscillating field of strength B_{STORM}. The rotating magnetic field is generated using two 30 cm long orthogonal saddle coils. The saddle coils are connected to a Keysight 33500B waveform generator supplying two synchronised channels with phase a shift of ±90°. The bias field is generated by the built-in Helmholtz coil of the Twinleaf shield. Elements of the setup are addressed using a Arduino Mega 2560 micro-controller unit (MCU).

Theoretical background

For simplicity let us consider a nuclear four-spin-system consisting of three spin-1/2 nuclei and a fourth spin-1 nucleus (deuterium for example). Two of the three spin-1/2 nuclei are assumed to be of isotopic type I, whereas the third spin-1/2 nucleus is of isotopic type S.

In solution the nuclei mutually interact by scalar spin-spin coupling terms

$$H_J = H_{II} + H_{IS} + H_{IX} + H_{SX}. \tag{1}$$

The scalar coupling Hamiltonians are explicitly given by

$$\begin{aligned} H_{II} &= 2\pi J_{12}\mathbf{I}_1 \cdot \mathbf{I}_2, & H_{IS} &= 2\pi J_{13}\mathbf{I}_1 \cdot \mathbf{S} + 2\pi J_{23}\mathbf{I}_2 \cdot \mathbf{S}, \\ H_{IX} &= 2\pi J_{14}\mathbf{I}_1 \cdot \mathbf{X} + 2\pi J_{24}\mathbf{I}_2 \cdot \mathbf{X}, & H_{IX} &= 2\pi J_{34}\mathbf{S} \cdot \mathbf{X}. \end{aligned} \tag{2}$$

The interaction of the nuclei with any external magnetic fields is described by the magnetic field Hamiltonian

$$H_M(t) = -\gamma_I \mathbf{B}(t) \cdot (\mathbf{I}_1 + \mathbf{I}_2) - \gamma_S \mathbf{B}(t) \cdot \mathbf{S} - \gamma_X \mathbf{B}(t) \cdot \mathbf{X}, \tag{3}$$

γ_I, γ_S and γ_X represent the magnetogyric ratio's of spins of isotopic type I, S and X, respectively.

In the current context the magnetic field Hamiltonian is a combination of a time-dependent rotating magnetic

field and a weak bias field along the laboratory frame z-axis

$$H_M = H_{\text{bias}} + H_{\text{rot}}(t). \tag{4}$$

The bias Hamiltonian is given by

$$\begin{aligned} H_{\text{bias}} &= -\gamma_I B_{\text{bias}}(I_{1z} + I_{2z}) - \gamma_S B_{\text{bias}} S_z - \gamma_X B_{\text{bias}} X_z \\ &= \omega_0^I (I_{1z} + I_{2z}) + \omega_0^S S_z + \omega_0^X X_z, \end{aligned} \tag{5}$$

whereas the rotating magnetic field Hamiltonian is given by

$$H_{\text{rot}}(t) = B_{\text{rot}} \cos(\Omega_{\text{rot}} t)(-\gamma_I(I_{1x} + I_{2x}) - \gamma_S S_x - \gamma_X X_x) - B_{\text{rot}} \sin(\Omega_{\text{rot}} t)(-\gamma_I(I_{1y} + I_{2y}) - \gamma_S S_y - \gamma_X X_y). \tag{6}$$

The total spin Hamiltonian is then a combination of scalar-coupling terms, the bias term and the rotating field contribution

$$H(t) = H_J + H_M(t) = H_J + H_{\text{bias}} + H_{\text{rot}}(t). \tag{7}$$

As discussed in reference 1 a STORM pulse is most easily analysed within a rotating frame along the laboratory frame z-axis with angular frequency Ω_{rot}

$$K_z(t) = \exp\{-i(I_{1z} + I_{2z} + S_z + X_z)\Omega_{\text{rot}} t\}. \tag{8}$$

The corresponding interaction frame Hamiltonian $\tilde{H}(t)$ is given by

$$\begin{aligned} \tilde{H} &= K_z(-t) H(t) K_z(t) + i\dot{K}_z(t) K_z(-t) \\ &= (\omega_0^I + \Omega_{\text{rot}})(I_{1z} + I_{2z}) + (\omega_0^S + \Omega_{\text{rot}}) S_z + (\omega_0^X + \Omega_{\text{rot}}) X_z + \omega_1^I (I_{1x} + I_{2x}) + \omega_1^S S_x + \omega_1^X X_x + H_J. \end{aligned} \tag{9}$$

STORM condition

Within the rotating frame the spins evolve under a new effective magnetic field B_{eff}. The coupling of the spins to effective field may be characterised by the effective nutation frequencies ω_{eff}^j

$$\omega_{\text{eff}}^j = \sqrt{(\omega_0^j + \Omega_{\text{rot}})^2 + (\omega_1^j)^2}, \tag{10}$$

and the polar angles θ_{eff}^j

$$\theta_{\text{eff}}^j = \text{arctan2}(\omega_0^j + \Omega_{\text{rot}}, \omega_1^j). \tag{11}$$

The polar angles describe the effective field direction with respect to the laboratory frame z-axis. This may be seen by expressing the rotating frame Hamiltonian as follows:

$$\tilde{H} = V H_{\text{eff}} V^\dagger + H_J, \tag{12}$$

where H_{eff} represents the effective field Hamiltonian in the absence of any scalar couplings

$$H_{\text{eff}} = \omega_{\text{eff}}^I (I_{1z} + I_{2z}) + \omega_{\text{eff}}^S S_z + \omega_{\text{eff}}^X X_z. \tag{13}$$

The transformation V is defined as a composite rotation along the laboratory frame y-axis of spins I, S and X

$$V = R_y^{12}(\theta_{\text{eff}}^I) R_y^3(\theta_{\text{eff}}^S) R_y^4(\theta_{\text{eff}}^X). \tag{14}$$

As discussed in reference 1 the efficiency of a STORM pulse is maximised by choosing the rotation frequency Ω_{rot} to be the root of the STORM condition C_{STORM}

$$C_{\text{STORM}} = \omega_{\text{eff}}^I - \omega_{\text{eff}}^S \pm 2\pi \left(J_{12} - \frac{J_{13} + J_{23}}{4} \cos(\theta_{\text{eff}}^I - \theta_{\text{eff}}^S) \right), \tag{15}$$

Table 1 List of various STORM conditions for [1-^{13}C, 2,3-d$_2$]-succinic acid found as a root of equation 15.

B_{bias}	B_1	$\omega^1_{STORM}/(2\pi)$	$\omega^2_{STORM}/(2\pi)$	B_{bias}	B_1	$\omega^1_{STORM}/(2\pi)$	$\omega^2_{STORM}/(2\pi)$
4 μT	2 μT	128 Hz	139 Hz	6 μT	2 μT	173 Hz	182 Hz
4 μT	4 μT	203 Hz	224 Hz	6 μT	4 μT	224 Hz	238 Hz
4 μT	6 μT	329 Hz	366 Hz	6 μT	6 μT	310 Hz	330 Hz

This particular angular frequency value is known as the STORM frequency ω_{STORM}

$$\Omega_{rot} = \omega_{STORM} \implies C_{STORM} = 0. \tag{16}$$

Deuterium decoupling

In general, an appropriate STORM frequency may be found for various strengths of the bias field, and the rotating magnetic field - albeit numerically. As a result there is a large freedom in choosing the amplitude of the bias field and the rotating field.

We utilise this freedom to decouple the X spin (deuterium) from the I spins during the STORM pulse. In particular, we may fix the bias field B_{bias} and choose the amplitude of the rotating field B_{STORM} in such a way that the effective magnetic fields of the I spins and the X spin within the rotating-frame are perpendicular to one another

$$\Delta\theta^{IX}_{eff} = \theta^{I}_{eff} - \theta^{S}_{eff} \stackrel{!}{=} \pi/2. \tag{17}$$

The resulting situation is then analogous to continuous-wave decoupling in conventional high-field NMR and suppresses the scalar couplings of the X spin to the I spins.

Table 2 STORM conditions for [1-^{13}C, 2,3-d$_2$]-succinic acid found as a root of equation 15 that also fulfill equation 17.

B_{bias}	B_1	$\omega_{STORM}/(2\pi)$	B_{bias}	B_1	$\omega_{STORM}/(2\pi)$
2 μT	1.2 μT	79 Hz	5 μT	3.2 μT	195 Hz
3 μT	1.9 μT	118 Hz	6 μT	3.9 μT	233 Hz
4 μT	2.5 μT	156 Hz	7 μT	4.6 μT	273 Hz

Suppression of coherent mixing with WOLF+STORM

We now consider the suppression of coherent singlet state mixing due to couplings between the I spins and the S spin. The suppression strategy may be subdivided into two steps.

We first consider the situation of a resonant STORM pulse. The bias field and the rotating magnetic field strength are assumed to be adjusted to ensure decoupling of the X spin as discussed above. For simplicity we then ignore the X spin and concentrate on the resulting three-spin-1/2 system. The scalar coupling Hamiltonian for the remainder of this section will thus be taken to be

$$H_J = H_{II} + H_{IS}, \tag{18}$$

whereas the magnetic field Hamiltonian is given by

$$H_M(t) = -\gamma_I \mathbf{B}(t)\cdot(\mathbf{I}_1 + \mathbf{I}_2) - \gamma_S \mathbf{B}(t)\cdot\mathbf{S}, \tag{19}$$

The discussion in reference 1 shows that the resonant STORM pulse isolates the following three states

$$|S_0\beta'\rangle = V|S_0\beta\rangle, \quad |T_0\beta'\rangle = V|T_0\beta\rangle, \quad |T_{-1}\alpha'\rangle = V|T_{-1}\alpha\rangle. \tag{20}$$

Under the STORM condition the states $|S_0\beta'\rangle$ and $|T_{-1}\alpha'\rangle$ are exactly degenerate and are subjected to coherent mixing. During the polarisation transfer step this is desirable as it enables fast generation of S spin magnetisation. However, during the bubbling period strong mixing of these states is undesirable as it favours the loss of singlet-order due to singlet-triplet mixing [2–4].

In a second step we aim to suppress the coherent mixing effects by adding an oscillating magnetic field along the

laboratory frame z-axis

$$H_{\mathrm{osc}}(t) = -B_{\mathrm{WOLF}}\cos(\Omega_{\mathrm{WOLF}}t)(\gamma_I(I_{1z}+I_{2z})+\gamma_S S_z) = \cos(\Omega_{\mathrm{WOLF}}t)(\omega^I_{\mathrm{WOLF}}(I_{1z}+I_{2z})+\omega^S_{\mathrm{WOLF}}S_z). \tag{21}$$

This is a particular convenient choice since the oscillating field Hamiltonian commutes with the rotation operator K_z generating the rotating frame of the STORM pulse

$$K_z(t)H_{\mathrm{osc}}(t)K_z(-t) = H_{\mathrm{osc}}(t). \tag{22}$$

The oscillating field Hamiltonian then simply adds to the interaction frame Hamiltonian

$$\tilde{H}(t) = H_J + \omega^I_1(I_{1x}+I_{2x}) + \omega^S_1 S_x + (\omega^I_0+\Omega_{\mathrm{rot}})(I_{1z}+I_{2z}) + (\omega^S_0+\Omega_{\mathrm{rot}})S_z + H_{\mathrm{osc}}(t). \tag{23}$$

To remove the time-dependence of the Hamiltonian in equation 23 we perform an additional interaction frame transformation defined by

$$Y(t) = \exp\left\{-i\int_0^t H_{\mathrm{osc}}(s)ds\right\}. \tag{24}$$

The resulting interaction frame Hamiltonain is given by

$$\tilde{\tilde{H}}(t) = Y(-t)\tilde{H}(t)Y(t) - H_{\mathrm{osc}}(t). \tag{25}$$

For sufficiently fast modulation frequencies Ω_{WOLF} we may average $\tilde{\tilde{H}}(t)$ over one period

$$\begin{aligned}\overline{H} &= \frac{\Omega_{\mathrm{WOLF}}}{2\pi}\int_0^{2\pi/\Omega_{\mathrm{WOLF}}}\tilde{\tilde{H}}(s)ds \\ &= (\omega^I_0+\Omega_{\mathrm{rot}})(I_{1z}+I_{2z}) + (\omega^S_0+\Omega_{\mathrm{rot}})S_z + J_0(\frac{\omega^I_{\mathrm{WOLF}}}{\Omega_{\mathrm{WOLF}}})\omega^I_1(I_{1x}+I_{2x}) + J_0(\frac{\omega^S_{\mathrm{WOLF}}}{\Omega_{\mathrm{WOLF}}})\omega^S_1 S_x \\ &+ H_{II} + \sum_{i=1}^{2} J_{i3}\{I_{iz}S_z + J_0(\frac{\omega^I_{\mathrm{WOLF}}-\omega^S_{\mathrm{WOLF}}}{\Omega_{\mathrm{WOLF}}})(I_{ix}S_x+I_{iy}S_y)\},\end{aligned} \tag{26}$$

where $J_0(x)$ is the 0'th Bessel function of the first kind. The presence of the oscillating z-field thus leads to a modification of the effective field strength

$$\mu^j_{\mathrm{eff}} = \sqrt{(\omega^j_0+\Omega_{\mathrm{rot}})^2 + \left(\omega^j_1 J_0\left(\frac{\omega^j_{\mathrm{WOLF}}}{\Omega_{\mathrm{WOLF}}}\right)\right)^2}, \tag{27}$$

and the direction

$$\phi^j_{\mathrm{eff}} = \mathrm{arctan2}(\omega^j_0+\Omega_{\mathrm{rot}}, \omega^j_1 J_0\left(\frac{\omega^j_{\mathrm{WOLF}}}{\Omega_{\mathrm{WOLF}}}\right)). \tag{28}$$

The matrix representation of $\overline{H}$ restricted to the manifold $M = \{|S_0\beta'\rangle, |T_0\beta'\rangle, |T_{-1}\alpha'\rangle\}$ is given by

$$[\overline{H}]_M = \begin{bmatrix} h_{11} & h_{12} & h_{13} \\ h_{12} & h_{22} & h_{23} \\ h_{13} & h_{23} & h_{33} \end{bmatrix} \tag{29}$$

where the individual elements are given by

$$
\begin{aligned}
h_{11} &= \frac{1}{2}\mu^S_{\text{eff}} - \frac{3}{2}\pi J_{12}, \quad h_{22} = \frac{1}{2}\mu^S_{\text{eff}} + \frac{1}{2}\pi J_{12}, \quad h_{33} = \mu^I_{\text{eff}} - \frac{1}{2}\mu^S_{\text{eff}} + \frac{1}{2}\pi J_{12}, \\
h_{12} &= \pi\Delta J\{\cos(\phi^I_{\text{eff}})\cos(\phi^S_{\text{eff}}) + J_0(\frac{\omega^I_{\text{WOLF}} - \omega^S_{\text{WOLF}}}{\Omega_{\text{WOLF}}})\sin(\phi^I_{\text{eff}})\sin(\phi^S_{\text{eff}})\}, \\
h_{13} &= -\frac{\pi}{\sqrt{2}}\Delta J\{J_0(\frac{\omega^I_{\text{WOLF}} - \omega^S_{\text{WOLF}}}{\Omega_{\text{WOLF}}})(1 + \cos(\phi^I_{\text{eff}})\cos(\phi^S_{\text{eff}})) + \sin(\phi^I_{\text{eff}})\sin(\phi^S_{\text{eff}})\}, \\
h_{23} &= \frac{\pi}{\sqrt{2}}\Sigma J\{J_0(\frac{\omega^I_{\text{WOLF}} - \omega^S_{\text{WOLF}}}{\Omega_{\text{WOLF}}})(1 + \cos(\phi^I_{\text{eff}})\cos(\phi^S_{\text{eff}})) + \sin(\phi^I_{\text{eff}})\sin(\phi^S_{\text{eff}})\}.
\end{aligned}
\tag{30}
$$

For simplicity we introduced the additional abbreviations

$$\Delta J = \frac{1}{2}(J_{13} - J_{23}), \quad \Sigma J = \frac{1}{2}(J_{13} + J_{23}). \tag{31}$$

The degree of coherent mixing may be quantified in terms of two mixing parameters ξ_{12} and ξ_{13}

$$\xi_{12} = |\frac{h_{12}}{h_{11} - h_{22}}|, \quad \xi_{13} = |\frac{h_{13}}{h_{11} - h_{33}}|. \tag{32}$$

Coherent mixing may now be efficiently suppressed by choosing the WOLF amplitude ω_{WOLF} and the WOLF frequency Ω_{WOLF} to minimise the sum of the mixing parameters

$$\min_{\omega_{\text{WOLF}},\Omega_{\text{WOLF}}} \xi_{12} + \xi_{13}, \tag{33}$$

which in general is easily solved numerically.

In an idealised situation we may assume that both ξ_{12} and ξ_{13} are sufficiently close to zero

$$\xi_{12} \simeq 0, \quad \xi_{13} \simeq 0. \tag{34}$$

We may then ignore the off-diagonal elements connecting the state $|S_0\beta'\rangle$ to $|T_0\beta'\rangle$ and $|T_{-1}\alpha'\rangle$ altogether. The restricted Hamiltonian $[\overline{H}]_M$ then takes the simplified form

$$[\overline{H}]_M \simeq \begin{bmatrix} h_{11} & 0 & 0 \\ 0 & h_{22} & h_{23} \\ 0 & h_{23} & h_{33} \end{bmatrix}, \tag{35}$$

indicating that the spin state $|S_0\beta'\rangle$ (similarly for $|S_0\alpha'\rangle$) is completely isolated from all other spin states of the system.

Let us now assume that the hydrogen gas has been fully enriched in its para spin-isomer so that all hydrogen molecules are found in their nuclear singlet state. The density operator for an individual reactant immediately after the hydrogenation reaction may then be expressed as follows

$$\rho(0) = |S_0\alpha\rangle\langle S_0\alpha| + |S_0\beta\rangle\langle S_0\beta| = |S_0\alpha'\rangle\langle S_0\alpha'| + |S_0\beta'\rangle\langle S_0\beta'|. \tag{36}$$

The second equality follows from the rotational invariance of the initial density operator. According to equation 35 the spin state $|S_0\beta'\rangle$ (similarly for $|S_0\alpha'\rangle$) only acquires an inconsequential phase factor under an idealised WOLF+STORM pulse. This in turn implies that the initial density operator $\rho(0)$ is invariant under the application of a WOLF+STORM pulse

$$U_{\text{W+S}}(t)\rho(0)U^\dagger_{\text{W+S}}(t) = \rho(0), \tag{37}$$

where $U_{\text{W+S}}(t)$ represents the WOLF+STORM propagator. This shows that the combination of a suitably chosen WOLF+STORM pulse stabilises nuclear singlet order irrespective of when the reactant has been hydrogenated, thus leading to a constructive accumulation of nuclear singlet order during the bubbling period.

To conclude we would like to point out that since the oscillating magnetic field introduces a small modification to

Table 3 Spin-spin coupling parameters for the effective 4-spin system aiming to approximate [1-^{13}C, 2,3-d$_2$]-succinic acid.

J_{12}	J_{13}	J_{23}	J_{14}	J_{34}
7.41 Hz	5.82 Hz	-7.15 Hz	4.00 Hz	-2.00 Hz

X spin decoupling condition through equations 27 and 28, there is in general a trade-off between optimal suppression of coherent mixing and decoupling.

Numerical search for WOLF+STORM conditions

We now outline a simple strategy to numerically search for WOLF and STORM frequencies compatible with heteronuclear decoupling and suppression of coherent singlet-triplet mixing effects. The corresponding Mathematica [?] notebook may be downloaded found from [?]. The optimization strategy chosen in this work may be summarised in a few simple steps:

1. Fix a value for B_{bias} and B_{STORM}, typically $B_{\text{STORM}} = 1/2B_{\text{bias}}$ is a good choice for any given B_{bias}.
2. Simultaneously minimize equations 15 and 17 to find a suitable STORM frequency ω_{STORM}.
3. With B_{bias}, B_{STORM} and ω_{STORM} derived from steps (1) and (2) minimize equation 33 over B_{WOLF} and Ω_{WOLF}. This will generate a suitable pair of parameters to suppress coherent singlet-triplet mixing effects.

In step (3) one may alternatively fix a reasonable value for B_{WOLF} and simply minimize over Ω_{WOLF}.

Monte-Carlo simulations

For the Monte-Carlo simulations we approximate the [1-^{13}C, 2,3-d$_2$]-succinic acid system by an effective 4 spin system consisting of two proton nuclei, one carbon nucleus and a single deuterium nucleus. Within this approximation the J-coupling Hamiltonian takes the form given by equation 1. To compensate for the absence of the second deuterium spin we increased the J-coupling values involving the deuterium by a factor of two compared to the values given in the main material. An overview of the coupling parameters is given in table 3.

The relaxation of the quadrupolar spin may be described by introducing a quadrupolar relaxation superoperator $\hat{\Gamma}_{\text{Q}}$. Following reference 5 the quadrupolar relaxation superoperator may be expressed as follows

$$\hat{\Gamma}_{\text{Q}} = -\frac{1}{5}(T_1^{\text{Q}})^{-1} \sum_{m=-2}^{+2} (-1)^m \hat{T}_{2m}^{\text{Q}}(X) \hat{T}_{2-m}^{\text{Q}}(X), \tag{38}$$

where T_1^{Q} represents the longitudinal relaxation time constant of the deuterium spin, which we estimated to be around $T_1^{\text{Q}} \sim 150\,\text{ms}$. The various $\hat{T}_{2m}^{\text{Q}}(X)$ represent commutation superoperators generated by a spherical tensor operator of rank 2 and z-projection value m for the deuterium spin. An explicit expression of these operators may be found in reference 5 for example.

We account for the relaxation of the protons spins by introducing a relaxation superoperator $\hat{\Gamma}_{\text{H}}$ approximating the effects of random magnetic field fluctuations experienced by the spins. The random field relaxation superoperator may be expressed as follows [5]:

$$\hat{\Gamma}_{\text{H}} = -\frac{17}{12}(T_1^{\text{H}})^{-1} \sum_{i,j=1}^{2} \sum_{m=-1}^{+1} \kappa_{ij} (-1)^m \hat{T}_{1m}^{\text{H}}(i) \hat{T}_{1-m}^{\text{H}}(j). \tag{39}$$

Here, T_1^{H} represents the longitudinal relaxation time constant of the proton spins and κ_{ij} describes the correlations between the randomly fluctuating fields experienced by proton i and proton j. The correlation coefficients may be expressed in terms of the relaxation times of the system as follows

$$\begin{aligned} \kappa_{11} &= \kappa_{22} = 1, \\ \kappa_{12} &= \kappa_{21} = 1 - \frac{3}{17}\frac{T_1^{\text{H}}}{T_S^{\text{H}}}, \end{aligned} \tag{40}$$

where T_S^{H} represents the nuclear singlet order decay time constant for the proton-pair. Both T_1^{H} and T_S^{H} where treated as fitting parameters. An explicit consideration of the relaxation of the carbon nucleus was found to be negligible

due to its comparably long relaxation time constants.

To combine coherent and incoherent contributions to the spin dynamics we introduce a coherent Liouvillian superoperator $\hat{L}_{\text{coh}}$

$$\hat{L}_{\text{coh}}(t) = -i\hat{H}_{\text{coh}}(t), \tag{41}$$

where the coherent commutation superoperator $\hat{H}_{\text{coh}}$ is given by

$$\hat{H}_{\text{coh}}(t) = \hat{H}_J + \hat{H}_M(t) \tag{42}$$

and defined by

$$\hat{H}_{\text{coh}}(t)\rho(t) = [H_{\text{coh}}(t), \rho(t)]. \tag{43}$$

The dynamics of the spin ensemble are then described by the Liouville-von-Neuman equation

$$\frac{d}{dt}\rho(t) = (\hat{L}_{\text{coh}}(t) + \hat{\Gamma}_{\text{Q}} + \hat{\Gamma}_{\text{H}})\rho(t). \tag{44}$$

To simulate the bubbling period of the experimental protocol we assume that the chemical dynamics of the reactant ([1-^{13}C, 2,3-d$_2$]-fumaric acid) effectively follow a unimolecular reaction scheme

$$\frac{d}{dt}[R(t)] = -k_{\text{eff}}[R(t)]. \tag{45}$$

The reaction times τ_r at which point [1-^{13}C, 2,3-d$_2$]-fumaric acid is being hydrogenated into [1-^{13}C, 2,3-d$_2$]-succinic acid are then distributed exponentially

$$\tau_r \sim k_{\text{eff}} \exp(-k_{\text{eff}} t), \tag{46}$$

where $\tau_{\text{eff}} = k_{\text{eff}}^{-1}$ represents the mean life time of the reactant.

We may proceed by generating a set of samples S according to 46

$$S = \{\tau_r^1, \tau_r^2, \ldots, \tau_r^N\}. \tag{47}$$

Each individual sample represents the fate of a single reactant during the bubbling period. For every individual member we split the bubbling period of duration τ_{B} into two parts

$$\tau_a^j = \tau_r^j, \quad \tau_b^j = \tau_{\text{B}} - \tau_r^j. \tag{48}$$

If $\tau_r^j > \tau_{\text{B}}$ no reaction occurs and the corresponding ensemble member does not contribute to the observed signal.

During τ_a^j the j'th ensemble member is still identified as [1-^{13}C, 2,3-d$_2$]-fumaric acid, its corresponding evolution may be ignored. At τ_b^j the j'th ensemble member has reacted with pH$_2$ to form [1-^{13}C, 2,3-d$_2$]-succinic acid. The density operator for the j'th ensemble member immediately after the reaction is given by

$$\rho^j(0) = \rho_{\text{pH}_2} \otimes \mathbb{1}_{S+X}, \tag{49}$$

where ρ_{pH_2} describes the state of the dissolved para-hydrogen gas and $\mathbb{1}_{S+X}$ represents the identity operator for a spin system formed by a single ^{13}C and H$_2$ spin.

Individual density operators $\rho^j(0)$ are then propagated in time for a duration τ_b^j assuming an effective [1-^{13}C, 2,3-d$_2$]-succinic acid spin system with the coupling parameters summarised in table 3. The magnetic field commutation superoprator in equation 42 consists of a static bias field in the case of protocol (b) and a WOLF+STORM pulse for protocol (c). The ensemble averaged density operator for the [1-^{13}C, 2,3-d$_2$]-succinic acid molecules after the bubbling period may then be approximated as follows

$$\rho(t) = \frac{1}{N}\sum_{j=1}^{N} \rho^j(t). \tag{50}$$

Any subsequent spin manipulations such as the polarization transfer step may then be simulated according to standard techniques.

Linear field sweep optimization

Time periods τ_T and τ_B for protocol (a) were optimized running a separate set of experiments. The results are shown in Fig. S2. The optimal values where found to be 90 ms and 7 s for τ_T and τ_B, respectively.

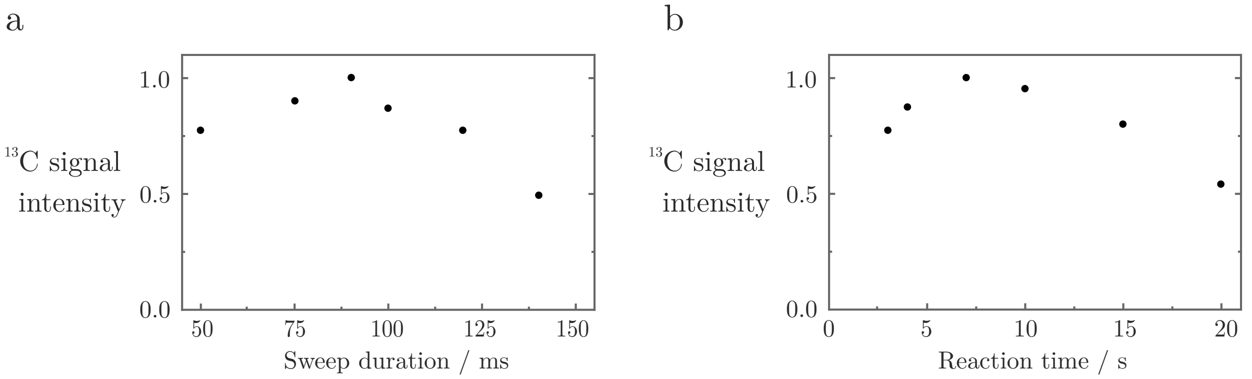

Figure S2 Signal intensity of hyperpolarized [1-^{13}C, 2,3-d_2]-succinic acid produced by hydrogenation with *para*-enriched hydrogen at static magnetic field of 56 μT followed by application of magnetic field sweep from 0 to 1 μT. (a) ^{13}C signal intensity as a function of sweep duration with reaction time fixed at 4 s. (b) ^{13}C signal intensity as a function of reaction time with linear sweep duration fixed at 90 ms. Data points are normalized to maximum signal.

Molar Polarization estimate

The reaction yield was estimated by acquiring ^{13}C NMR spectra of the samples prior to chemical reaction with *para*-hydrogen and after the conclusion of the reaction (Fig. S3). The chemical shift of [1-^{13}C, 2,3-d_2]-succinic acid and [1-^{13}C, 2,3-d_2]-fumaric acid are distinctively different. This allows estimating the concentration of [1-^{13}C, 2,3-d_2]-succinic acid after experiment. Spectra suggest a small amount of [1-^{13}C, 2,3-d_2]-fumaric acid being present after the reaction. The total yield was estimated to be 98-99% which implies a final [1-^{13}C, 2,3-d_2]-succinic acid concentration of 49 ± 1 mM. The molar polarization is obtained as a product of the approximate reaction yield and the achieved polarization level. This leads to an estimated molar polarization of $6.12\% \times (49 \pm 1)$ mM $= 3.00 \pm 0.06$ mM as indicated in the main part of the manuscript.

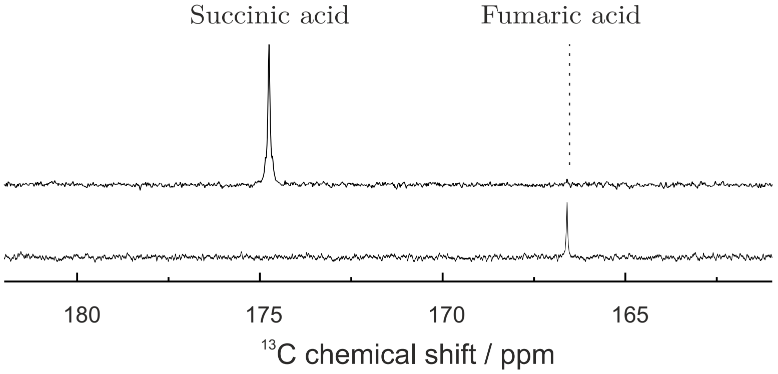

Figure S3 ^{13}C spectra of the solutions samples before and after PHIP experiments. Top - ^{13}C-spectrum after the conclusion of the hydrogenation reaction, the spectrum has been averaged over 32 transients. Bottom - ^{13}C-spectrum of the precursor solution prior to hydrogenation, the spectrum represents a single transient. The chemical shifts of [1-^{13}C, 2,3-d_2]-succinic acid and [1-^{13}C, 2,3-d_2]-fumaric acid are indicated above. Vertical axis have been scaled to match noise levels.

References

1 L. Dagys, C. Bengs, *Phys. Chem. Chem. Phys.* **2022**.

2 D. A. Markelov, V. P. Kozinenko, S. Knecht, A. S. Kiryutin, A. V. Yurkovskaya, K. L. Ivanov, *Phys. Chem. Chem. Phys.* **2021**, *23*, 20936–20944.

3 L. Dagys, B. Ripka, M. Leutzsch, G. A. I. Moustafa, J. Eills, J. F. P. Colell, M. H. Levitt, *Magn. Reson.* **2020**, *1*, 175–186.

4 S. J. Barker, L. Dagys, W. Hale, B. Ripka, J. Eills, M. Sharma, M. H. Levitt, M. Utz, *Anal. Chem.* **2022**, *94*, 3260–3267.

5 S. J. Elliott, C. Bengs, L. J. Brown, J. T. Hill-Cousins, D. J. O'Leary, G. Pileio, M. H. Levitt, *J Chem Phys* **2019**, *150*, 064315.
